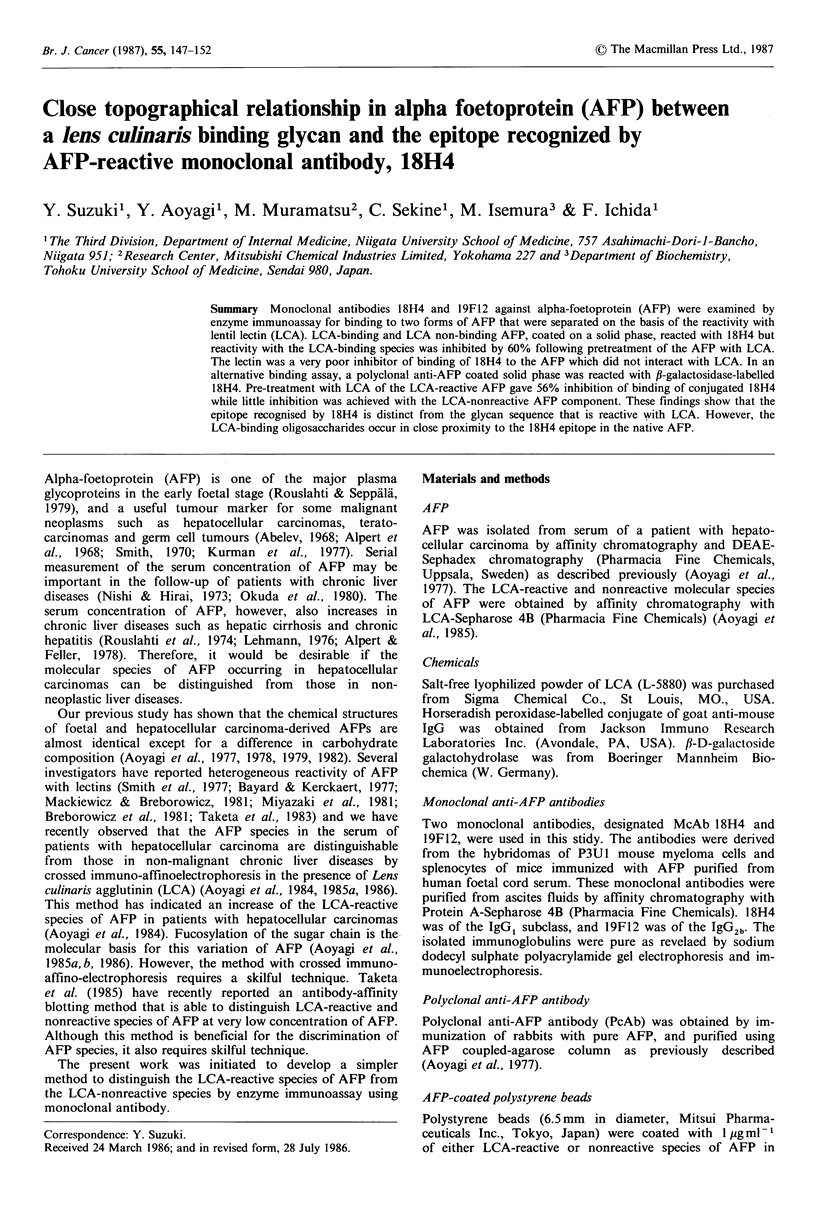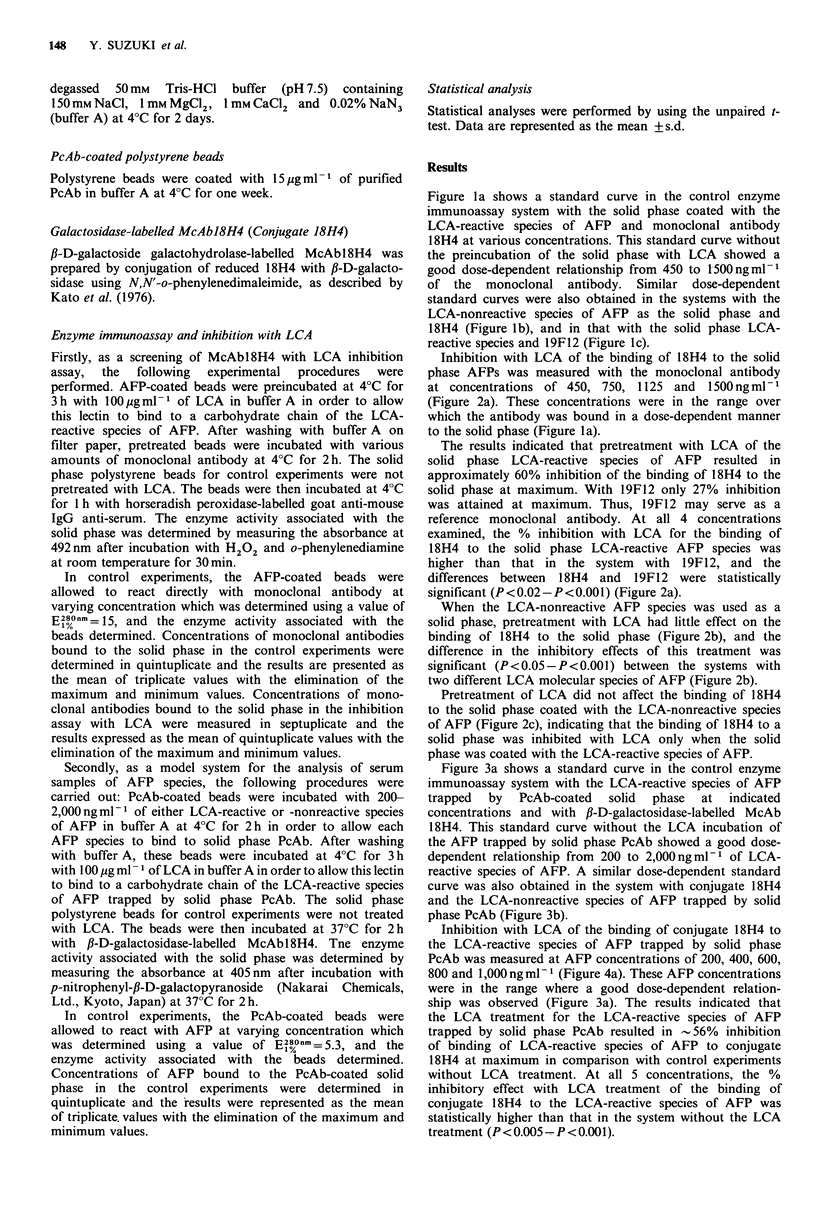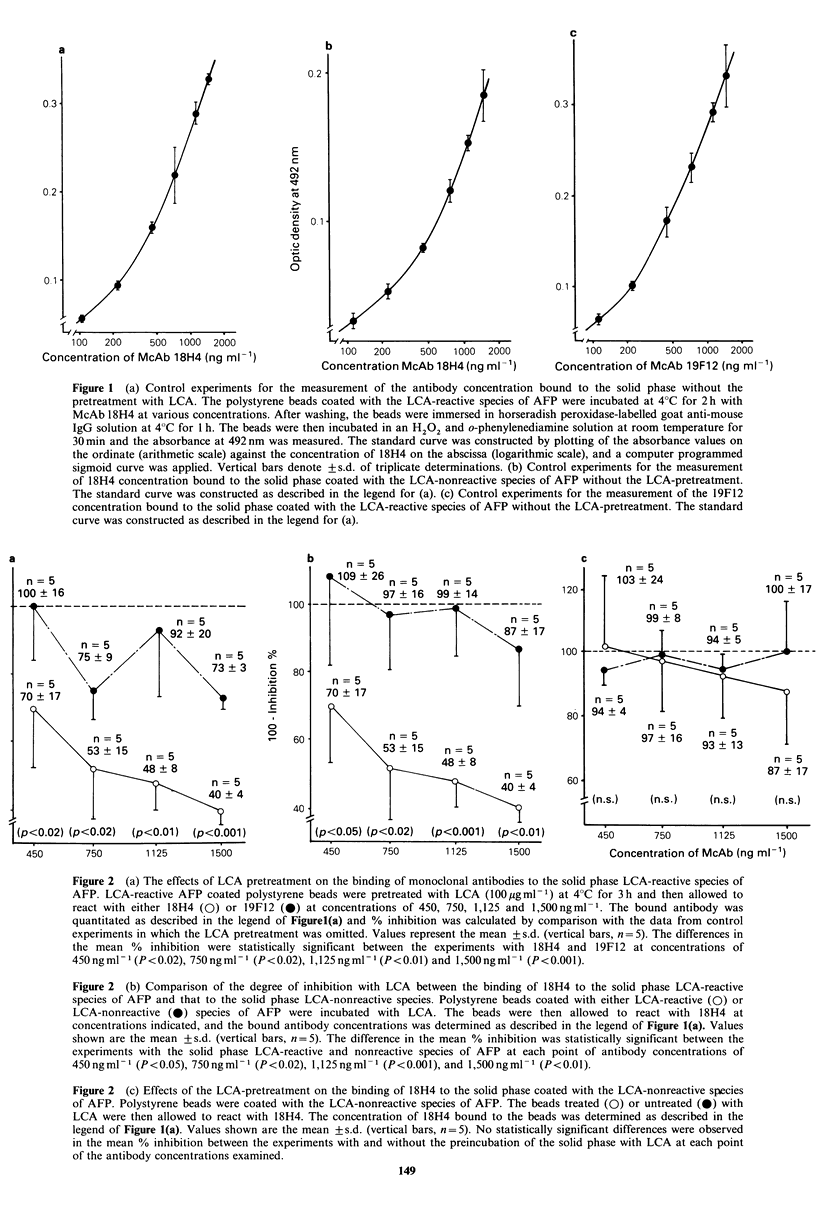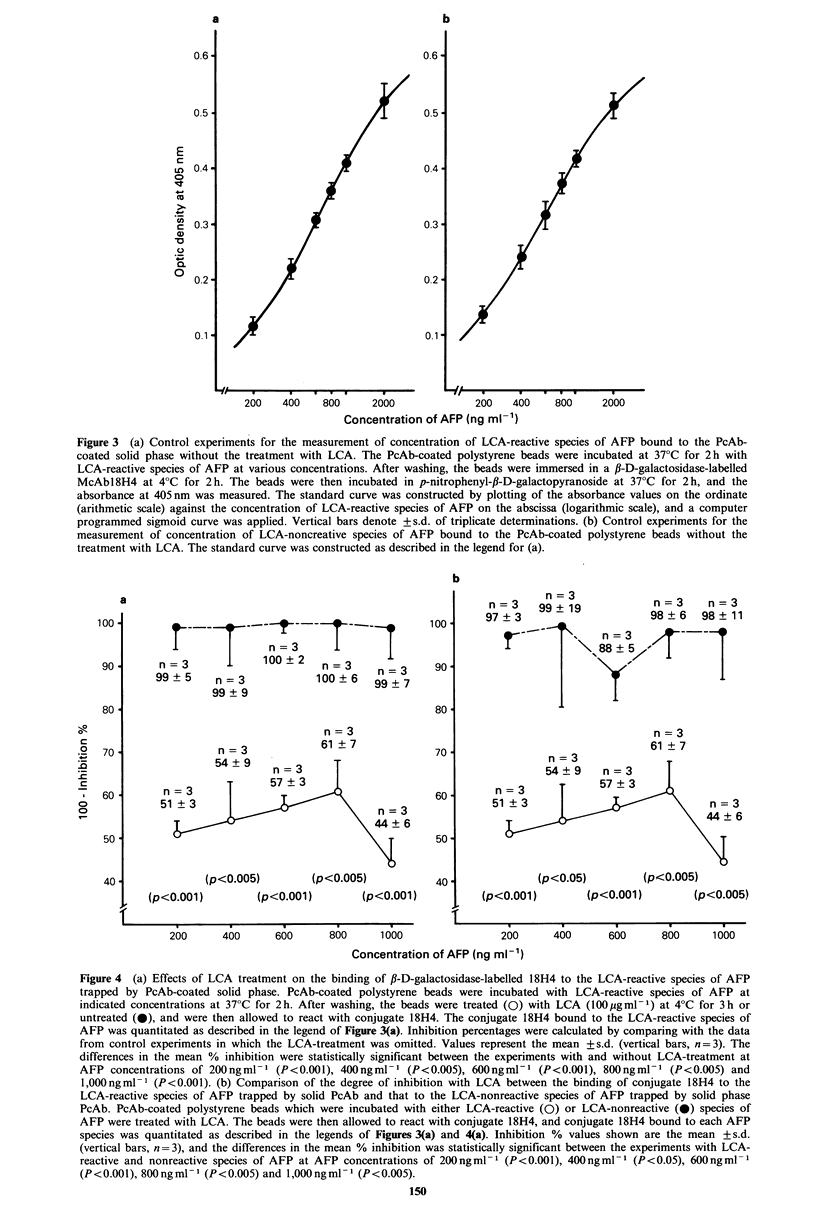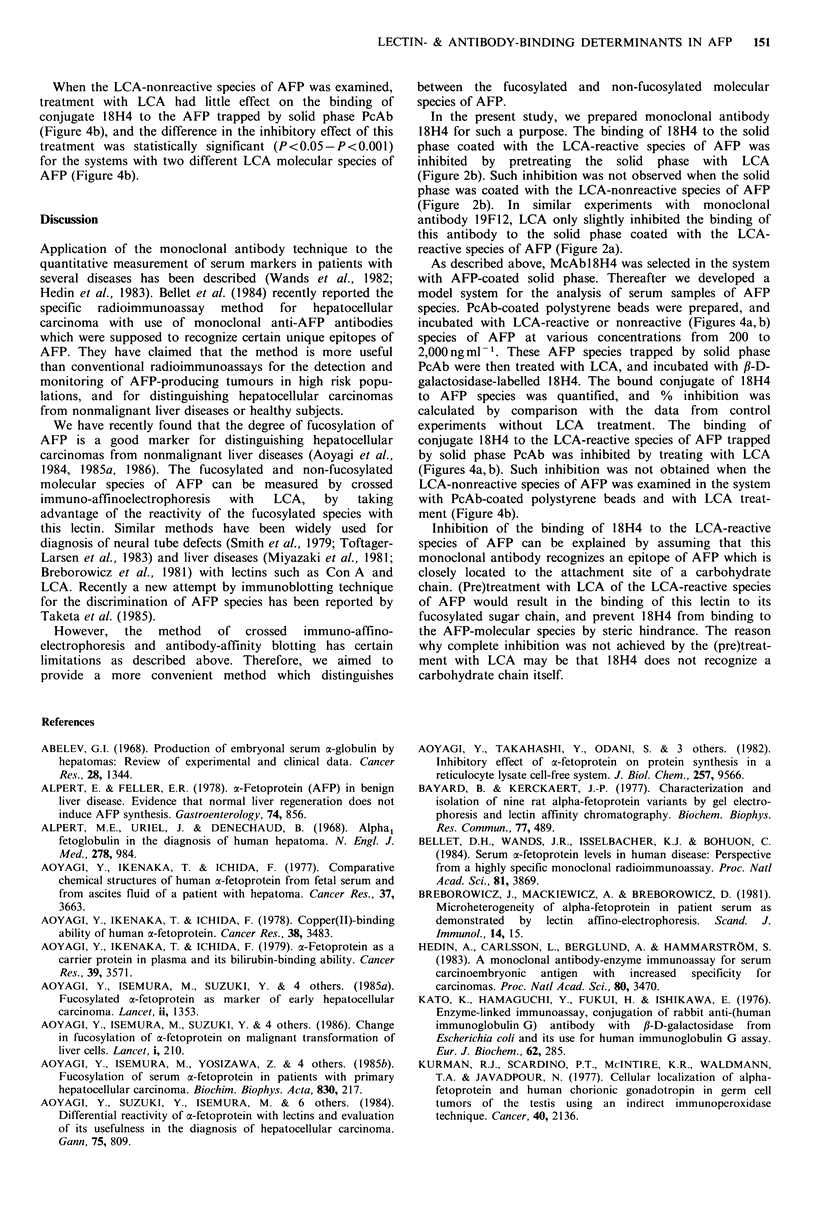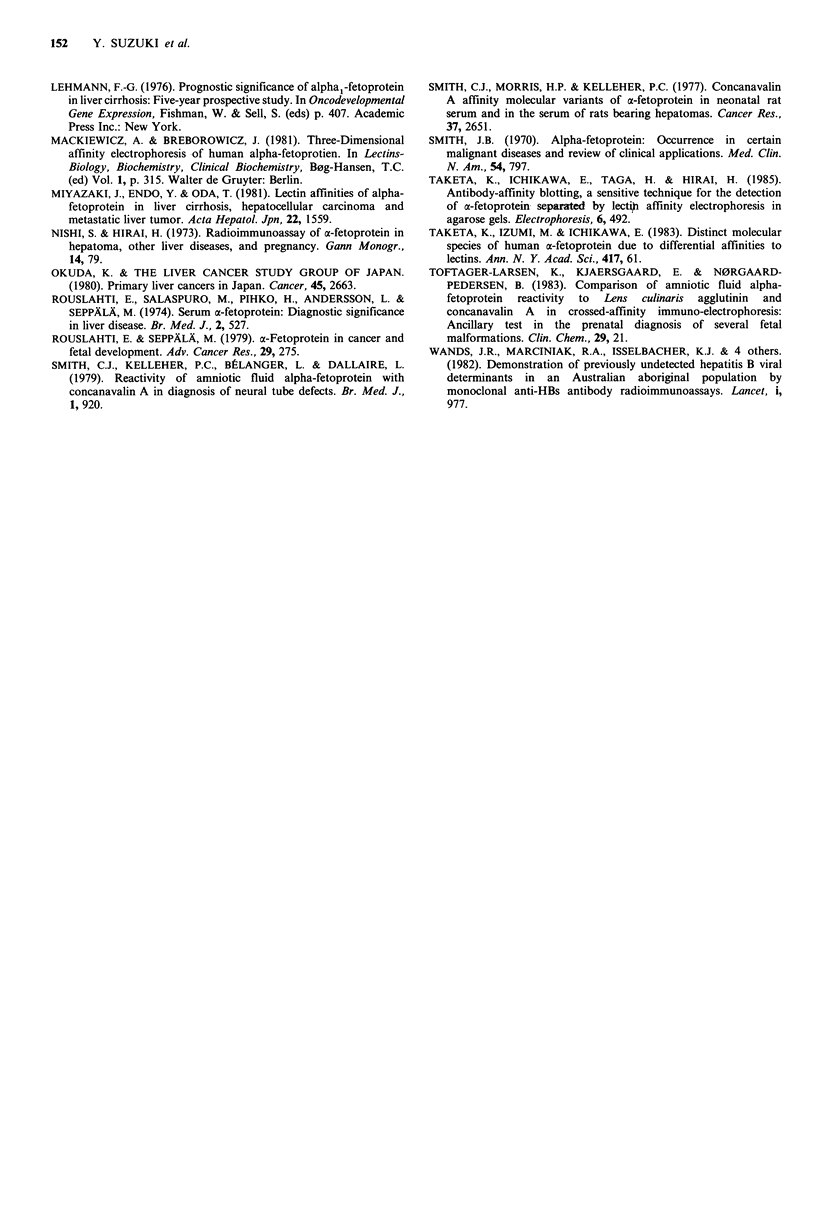# Close topographical relationship in alpha foetoprotein (AFP) between a lens culinaris binding glycan and the epitope recognized by AFP-reactive monoclonal antibody, 18H4.

**DOI:** 10.1038/bjc.1987.30

**Published:** 1987-02

**Authors:** Y. Suzuki, Y. Aoyagi, M. Muramatsu, C. Sekine, M. Isemura, F. Ichida

## Abstract

Monoclonal antibodies 18H4 and 19F12 against alpha-foetoprotein (AFP) were examined by enzyme immunoassay for binding to two forms of AFP that were separated on the basis of the reactivity with lentil lectin (LCA). LCA-binding and LCA non-binding AFP, coated on a solid phase, reacted with 18H4 but reactivity with the LCA-binding species was inhibited by 60% following pretreatment of the AFP with LCA. The lectin was a very poor inhibitor of binding of 18H4 to the AFP which did not interact with LCA. In an alternative binding assay, a polyclonal anti-AFP coated solid phase was reacted with beta-galactosidase-labelled 18H4. Pre-treatment with LCA of the LCA-reactive AFP gave 56% inhibition of binding of conjugated 18H4 while little inhibition was achieved with the LCA-nonreactive AFP component. These findings show that the epitope recognised by 18H4 is distinct from the glycan sequence that is reactive with LCA. However, the LCA-binding oligosaccharides occur in close proximity to the 18H4 epitope in the native AFP.


*Br. J. Cancer* (1987), **55**, 147–152 © The Macmillan Press Ltd., 1987

### Close topographical relationship in alpha foetoprotein (AFP) between a *lens culinaris* binding glycan and the epitope recognized by AFP-reactive monoclonal antibody, 18H4

Y. Suzuki[1], Y. Aoyagi[1], M. Muramatsu[2], C. Sekine[1], M. Isemura[3] & F. Ichida[1]

[1] *The Third Division, Department of Internal Medicine, Niigata University School of Medicine, 757 Asahimachi-Dori-1-Bancho, Niigata 951;* [2] *Research Center, Mitsubishi Chemical Industries Limited, Yokohama 227 and* [3] *Department of Biochemistry, Tohoku University School of Medicine, Sendai 980, Japan.*

**Summary** Monoclonal antibodies 18H4 and 19F12 against alpha-foetoprotein (AFP) were examined by enzyme immunoassay for binding to two forms of AFP that were separated on the basis of the reactivity with lentil lectin (LCA). LCA-binding and LCA non-binding AFP, coated on a solid phase, reacted with 18H4 but reactivity with the LCA-binding species was inhibited by 60% following pretreatment of the AFP with LCA. The lectin was a very poor inhibitor of binding of 18H4 to the AFP which did not interact with LCA. In an alternative binding assay, a polyclonal anti-AFP coated solid phase was reacted with β-galactosidase-labelled 18H4. Pre-treatment with LCA of the LCA-reactive AFP gave 56% inhibition of binding of conjugated 18H4 while little inhibition was achieved with the LCA-nonreactive AFP component. These findings show that the epitope recognised by 18H4 is distinct from the glycan sequence that is reactive with LCA. However, the LCA-binding oligosaccharides occur in close proximity to the 18H4 epitope in the native AFP.

Alpha-foetoprotein (AFP) is one of the major plasma glycoproteins in the early foetal stage (Rouslahti & Seppälä, 1979), and a useful tumour marker for some malignant neoplasms such as hepatocellular carcinomas, teratocarcinomas and germ cell tumours (Abelev, 1968; Alpert *et al.*, 1968; Smith, 1970; Kurman *et al.*, 1977). Serial measurement of the serum concentration of AFP may be important in the follow-up of patients with chronic liver diseases (Nishi & Hirai, 1973; Okuda *et al.*, 1980). The serum concentration of AFP, however, also increases in chronic liver diseases such as hepatic cirrhosis and chronic hepatitis (Rouslahti *et al.*, 1974; Lehmann, 1976; Alpert & Feller, 1978). Therefore, it would be desirable if the molecular species of AFP occurring in hepatocellular carcinomas can be distinguished from those in non-neoplastic liver diseases.

Our previous study has shown that the chemical structures of foetal and hepatocellular carcinoma-derived AFPs are almost identical except for a difference in carbohydrate composition (Aoyagi *et al.*, 1977, 1978, 1979, 1982). Several investigators have reported heterogeneous reactivity of AFP with lectins (Smith *et al.*, 1977; Bayard & Kerckaert, 1977; Mackiewicz & Breborowicz, 1981; Miyazaki *et al.*, 1981; Breborowicz *et al.*, 1981; Taketa *et al.*, 1983) and we have recently observed that the AFP species in the serum of patients with hepatocellular carcinoma are distinguishable from those in non-malignant chronic liver diseases by crossed immuno-affinoelectrophoresis in the presence of *Lens culinaris* agglutinin (LCA) (Aoyagi *et al.*, 1984, 1985*a*, 1986). This method has indicated an increase of the LCA-reactive species of AFP in patients with hepatocellular carcinomas (Aoyagi *et al.*, 1984). Fucosylation of the sugar chain is the molecular basis for this variation of AFP (Aoyagi *et al.*, 1985*a*, *b*, 1986). However, the method with crossed immuno-affino-electrophoresis requires a skilful technique. Taketa *et al.* (1985) have recently reported an antibody-affinity blotting method that is able to distinguish LCA-reactive and nonreactive species of AFP at very low concentration of AFP. Although this method is beneficial for the discrimination of AFP species, it also requires skilful technique.

The present work was initiated to develop a simpler method to distinguish the LCA-reactive species of AFP from the LCA-nonreactive species by enzyme immunoassay using monoclonal antibody.

Correspondence: Y. Suzuki.
Received 24 March 1986; and in revised form, 28 July 1986.

### Materials and methods

### *AFP*

AFP was isolated from serum of a patient with hepatocellular carcinoma by affinity chromatography and DEAE-Sephadex chromatography (Pharmacia Fine Chemicals, Uppsala, Sweden) as described previously (Aoyagi *et al.*, 1977). The LCA-reactive and nonreactive molecular species of AFP were obtained by affinity chromatography with LCA-Sepharose 4B (Pharmacia Fine Chemicals) (Aoyagi *et al.*, 1985).

### *Chemicals*

Salt-free lyophilized powder of LCA (L-5880) was purchased from Sigma Chemical Co., St Louis, MO., USA. Horseradish peroxidase-labelled conjugate of goat anti-mouse IgG was obtained from Jackson Immuno Research Laboratories Inc. (Avondale, PA, USA). β-D-galactoside galactohydrolase was from Boeringer Mannheim Biochemica (W. Germany).

### *Monoclonal anti-AFP antibodies*

Two monoclonal antibodies, designated McAb 18H4 and 19F12, were used in this stidy. The antibodies were derived from the hybridomas of P3U1 mouse myeloma cells and splenocytes of mice immunized with AFP purified from human foetal cord serum. These monoclonal antibodies were purified from ascites fluids by affinity chromatography with Protein A-Sepharose 4B (Pharmacia Fine Chemicals). 18H4 was of the $IgG_1$ subclass, and 19F12 was of the $IgG_{2b}$. The isolated immunoglobulins were pure as revelaed by sodium dodecyl sulphate polyacrylamide gel electrophoresis and immunoelectrophoresis.

### *Polyclonal anti-AFP antibody*

Polyclonal anti-AFP antibody (PcAb) was obtained by immunization of rabbits with pure AFP, and purified using AFP coupled-agarose column as previously described (Aoyagi *et al.*, 1977).

### *AFP-coated polystyrene beads*

Polystyrene beads (6.5 mm in diameter, Mitsui Pharmaceuticals Inc., Tokyo, Japan) were coated with $1\,\mu g\,ml^{-1}$ of either LCA-reactive or nonreactive species of AFP in degassed 50 mM Tris-HCl buffer (pH 7.5) containing 150 mM NaCl, 1 mM $MgCl_2$, 1 mM $CaCl_2$ and 0.02% $NaN_3$ (buffer A) at 4°C for 2 days.

*PcAb-coated polystyrene beads*

Polystyrene beads were coated with 15 $\mu$g ml$^{-1}$ of purified PcAb in buffer A at 4°C for one week.

*Galactosidase-labelled McAb18H4 (Conjugate 18H4)*

$\beta$-D-galactoside galactohydrolase-labelled McAb18H4 was prepared by conjugation of reduced 18H4 with $\beta$-D-galactosidase using *N,N'-o*-phenylenedimaleimide, as described by Kato *et al.* (1976).

*Enzyme immunoassay and inhibition with LCA*

Firstly, as a screening of McAb18H4 with LCA inhibition assay, the following experimental procedures were performed. AFP-coated beads were preincubated at 4°C for 3 h with 100 $\mu$g ml$^{-1}$ of LCA in buffer A in order to allow this lectin to bind to a carbohydrate chain of the LCA-reactive species of AFP. After washing with buffer A on filter paper, pretreated beads were incubated with various amounts of monoclonal antibody at 4°C for 2 h. The solid phase polystyrene beads for control experiments were not pretreated with LCA. The beads were then incubated at 4°C for 1 h with horseradish peroxidase-labelled goat anti-mouse IgG anti-serum. The enzyme activity associated with the solid phase was determined by measuring the absorbance at 492 nm after incubation with $H_2O_2$ and *o*-phenylenediamine at room temperature for 30 min.

In control experiments, the AFP-coated beads were allowed to react directly with monoclonal antibody at varying concentration which was determined using a value of $E_{1\%}^{280nm} = 15$, and the enzyme activity associated with the beads determined. Concentrations of monoclonal antibodies bound to the solid phase in the control experiments were determined in quintuplicate and the results are presented as the mean of triplicate values with the elimination of the maximum and minimum values. Concentrations of monoclonal antibodies bound to the solid phase in the inhibition assay with LCA were measured in septuplicate and the results expressed as the mean of quintuplicate values with the elimination of the maximum and minimum values.

Secondly, as a model system for the analysis of serum samples of AFP species, the following procedures were carried out: PcAb-coated beads were incubated with 200–2,000 ng ml$^{-1}$ of either LCA-reactive or -nonreactive species of AFP in buffer A at 4°C for 2 h in order to allow each AFP species to bind to solid phase PcAb. After washing with buffer A, these beads were incubated at 4°C for 3 h with 100 $\mu$g ml$^{-1}$ of LCA in buffer A in order to allow this lectin to bind to a carbohydrate chain of the LCA-reactive species of AFP trapped by solid phase PcAb. The solid phase polystyrene beads for control experiments were not treated with LCA. The beads were then incubated at 37°C for 2 h with $\beta$-D-galactosidase-labelled McAb18H4. Tne enzyme activity associated with the solid phase was determined by measuring the absorbance at 405 nm after incubation with *p*-nitrophenyl-$\beta$-D-galactopyranoside (Nakarai Chemicals, Ltd., Kyoto, Japan) at 37°C for 2 h.

In control experiments, the PcAb-coated beads were allowed to react with AFP at varying concentration which was determined using a value of $E_{1\%}^{280nm} = 5.3$, and the enzyme activity associated with the beads determined. Concentrations of AFP bound to the PcAb-coated solid phase in the control experiments were determined in quintuplicate and the results were represented as the mean of triplicate values with the elimination of the maximum and minimum values.

*Statistical analysis*

Statistical analyses were performed by using the unpaired *t*-test. Data are represented as the mean ±s.d.

### Results

Figure 1a shows a standard curve in the control enzyme immunoassay system with the solid phase coated with the LCA-reactive species of AFP and monoclonal antibody 18H4 at various concentrations. This standard curve without the preincubation of the solid phase with LCA showed a good dose-dependent relationship from 450 to 1500 ng ml$^{-1}$ of the monoclonal antibody. Similar dose-dependent standard curves were also obtained in the systems with the LCA-nonreactive species of AFP as the solid phase and 18H4 (Figure 1b), and in that with the solid phase LCA-reactive species and 19F12 (Figure 1c).

Inhibition with LCA of the binding of 18H4 to the solid phase AFPs was measured with the monoclonal antibody at concentrations of 450, 750, 1125 and 1500 ng ml$^{-1}$ (Figure 2a). These concentrations were in the range over which the antibody was bound in a dose-dependent manner to the solid phase (Figure 1a).

The results indicated that pretreatment with LCA of the solid phase LCA-reactive species of AFP resulted in approximately 60% inhibition of the binding of 18H4 to the solid phase at maximum. With 19F12 only 27% inhibition was attained at maximum. Thus, 19F12 may serve as a reference monoclonal antibody. At all 4 concentrations examined, the % inhibition with LCA for the binding of 18H4 to the solid phase LCA-reactive AFP species was higher than that in the system with 19F12, and the differences between 18H4 and 19F12 were statistically significant ($P<0.02-P<0.001$) (Figure 2a).

When the LCA-nonreactive AFP species was used as a solid phase, pretreatment with LCA had little effect on the binding of 18H4 to the solid phase (Figure 2b), and the difference in the inhibitory effects of this treatment was significant ($P<0.05-P<0.001$) between the systems with two different LCA molecular species of AFP (Figure 2b).

Pretreatment of LCA did not affect the binding of 18H4 to the solid phase coated with the LCA-nonreactive species of AFP (Figure 2c), indicating that the binding of 18H4 to a solid phase was inhibited with LCA only when the solid phase was coated with the LCA-reactive species of AFP.

Figure 3a shows a standard curve in the control enzyme immunoassay system with the LCA-reactive species of AFP trapped by PcAb-coated solid phase at indicated concentrations and with $\beta$-D-galactosidase-labelled McAb 18H4. This standard curve without the LCA incubation of the AFP trapped by solid phase PcAb showed a good dose-dependent relationship from 200 to 2,000 ng ml$^{-1}$ of LCA-reactive species of AFP. A similar dose-dependent standard curve was also obtained in the system with conjugate 18H4 and the LCA-nonreactive species of AFP trapped by solid phase PcAb (Figure 3b).

Inhibition with LCA of the binding of conjugate 18H4 to the LCA-reactive species of AFP trapped by solid phase PcAb was measured at AFP concentrations of 200, 400, 600, 800 and 1,000 ng ml$^{-1}$ (Figure 4a). These AFP concentrations were in the range where a good dose-dependent relationship was observed (Figure 3a). The results indicated that the LCA treatment for the LCA-reactive species of AFP trapped by solid phase PcAb resulted in ~56% inhibition of binding of LCA-reactive species of AFP to conjugate 18H4 at maximum in comparison with control experiments without LCA treatment. At all 5 concentrations, the % inhibitory effect with LCA treatment of the binding of conjugate 18H4 to the LCA-reactive species of AFP was statistically higher than that in the system without the LCA treatment ($P<0.005-P<0.001$).

**Figure 1** (a) Control experiments for the measurement of the antibody concentration bound to the solid phase without the pretreatment with LCA. The polystyrene beads coated with the LCA-reactive species of AFP were incubated at 4°C for 2 h with McAb 18H4 at various concentrations. After washing, the beads were immersed in horseradish peroxidase-labelled goat anti-mouse IgG solution at 4°C for 1 h. The beads were then incubated in an $H_2O_2$ and *o*-phenylenediamine solution at room temperature for 30 min and the absorbance at 492 nm was measured. The standard curve was constructed by plotting of the absorbance values on the ordinate (arithmetic scale) against the concentration of 18H4 on the abscissa (logarithmic scale), and a computer programmed sigmoid curve was applied. Vertical bars denote ±s.d. of triplicate determinations. (b) Control experiments for the measurement of 18H4 concentration bound to the solid phase coated with the LCA-nonreactive species of AFP without the LCA-pretreatment. The standard curve was constructed as described in the legend for (a). (c) Control experiments for the measurement of the 19F12 concentration bound to the solid phase coated with the LCA-reactive species of AFP without the LCA-pretreatment. The standard curve was constructed as described in the legend for (a).

**Figure 2** (a) The effects of LCA pretreatment on the binding of monoclonal antibodies to the solid phase LCA-reactive species of AFP. LCA-reactive AFP coated polystyrene beads were pretreated with LCA (100 μg ml$^{-1}$) at 4°C for 3 h and then allowed to react with either 18H4 (○) or 19F12 (●) at concentrations of 450, 750, 1,125 and 1,500 ng ml$^{-1}$. The bound antibody was quantitated as described in the legend of **Figure1(a)** and % inhibition was calculated by comparison with the data from control experiments in which the LCA pretreatment was omitted. Values represent the mean ±s.d. (vertical bars, $n = 5$). The differences in the mean % inhibition were statistically significant between the experiments with 18H4 and 19F12 at concentrations of 450 ng ml$^{-1}$ ($P < 0.02$), 750 ng ml$^{-1}$ ($P < 0.02$), 1,125 ng ml$^{-1}$ ($P < 0.01$) and 1,500 ng ml$^{-1}$ ($P < 0.001$).

**Figure 2** (b) Comparison of the degree of inhibition with LCA between the binding of 18H4 to the solid phase LCA-reactive species of AFP and that to the solid phase LCA-nonreactive species. Polystyrene beads coated with either LCA-reactive (○) or LCA-nonreactive (●) species of AFP were incubated with LCA. The beads were then allowed to react with 18H4 at concentrations indicated, and the bound antibody concentrations was determined as described in the legend of **Figure 1(a)**. Values shown are the mean ±s.d. (vertical bars, $n = 5$). The difference in the mean % inhibition was statistically significant between the experiments with the solid phase LCA-reactive and nonreactive species of AFP at each point of antibody concentrations of 450 ng ml$^{-1}$ ($P < 0.05$), 750 ng ml$^{-1}$ ($P < 0.02$), 1,125 ng ml$^{-1}$ ($P < 0.001$), and 1,500 ng ml$^{-1}$ ($P < 0.01$).

**Figure 2** (c) Effects of the LCA-pretreatment on the binding of 18H4 to the solid phase coated with the LCA-nonreactive species of AFP. Polystyrene beads were coated with the LCA-nonreactive species of AFP. The beads treated (○) or untreated (●) with LCA were then allowed to react with 18H4. The concentration of 18H4 bound to the beads was determined as described in the legend of **Figure 1(a)**. Values shown are the mean ±s.d. (vertical bars, $n = 5$). No statistically significant differences were observed in the mean % inhibition between the experiments with and without the preincubation of the solid phase with LCA at each point of the antibody concentrations examined.

**Figure 3** (a) Control experiments for the measurement of concentration of LCA-reactive species of AFP bound to the PcAb-coated solid phase without the treatment with LCA. The PcAb-coated polystyrene beads were incubated at 37°C for 2 h with LCA-reactive species of AFP at various concentrations. After washing, the beads were immersed in a $\beta$-D-galactosidase-labelled McAb18H4 at 4°C for 2 h. The beads were then incubated in *p*-nitrophenyl-$\beta$-D-galactopyranoside at 37°C for 2 h, and the absorbance at 405 nm was measured. The standard curve was constructed by plotting of the absorbance values on the ordinate (arithmetic scale) against the concentration of LCA-reactive species of AFP on the abscissa (logarithmic scale), and a computer programmed sigmoid curve was applied. Vertical bars denote $\pm$s.d. of triplicate determinations. (b) Control experiments for the measurement of concentration of LCA-noncreative species of AFP bound to the PcAb-coated polystyrene beads without the treatment with LCA. The standard curve was constructed as described in the legend for (a).

**Figure 4** (a) Effects of LCA treatment on the binding of $\beta$-D-galactosidase-labelled 18H4 to the LCA-reactive species of AFP trapped by PcAb-coated solid phase. PcAb-coated polystyrene beads were incubated with LCA-reactive species of AFP at indicated concentrations at 37°C for 2 h. After washing, the beads were treated (○) with LCA (100 $\mu$g ml$^{-1}$) at 4°C for 3 h or untreated (●), and were then allowed to react with conjugate 18H4. The conjugate 18H4 bound to the LCA-reactive species of AFP was quantitated as described in the legend of **Figure 3(a)**. Inhibition percentages were calculated by comparing with the data from control experiments in which the LCA-treatment was omitted. Values represent the mean $\pm$s.d. (vertical bars, $n=3$). The differences in the mean % inhibition were statistically significant between the experiments with and without LCA-treatment at AFP concentrations of 200 ng ml$^{-1}$ ($P<0.001$), 400 ng ml$^{-1}$ ($P<0.005$), 600 ng ml$^{-1}$ ($P<0.001$), 800 ng ml$^{-1}$ ($P<0.005$) and 1,000 ng ml$^{-1}$ ($P<0.001$). (b) Comparison of the degree of inhibition with LCA between the binding of conjugate 18H4 to the LCA-reactive species of AFP trapped by solid PcAb and that to the LCA-nonreactive species of AFP trapped by solid phase PcAb. PcAb-coated polystyrene beads which were incubated with either LCA-reactive (○) or LCA-nonreactive (●) species of AFP were treated with LCA. The beads were then allowed to react with conjugate 18H4, and conjugate 18H4 bound to each AFP species was quantitated as described in the legends of **Figures 3(a)** and **4(a)**. Inhibition % values shown are the mean $\pm$s.d. (vertical bars, $n=3$), and the differences in the mean % inhibition was statistically significant between the experiments with LCA-reactive and nonreactive species of AFP at AFP concentrations of 200 ng ml$^{-1}$ ($P<0.001$), 400 ng ml$^{-1}$ ($P<0.05$), 600 ng ml$^{-1}$ ($P<0.001$), 800 ng ml$^{-1}$ ($P<0.005$) and 1,000 ng ml$^{-1}$ ($P<0.005$).

When the LCA-nonreactive species of AFP was examined, treatment with LCA had little effect on the binding of conjugate 18H4 to the AFP trapped by solid phase PcAb (Figure 4b), and the difference in the inhibitory effect of this treatment was statistically significant ($P<0.05-P<0.001$) for the systems with two different LCA molecular species of AFP (Figure 4b).

### Discussion

Application of the monoclonal antibody technique to the quantitative measurement of serum markers in patients with several diseases has been described (Wands *et al.*, 1982; Hedin *et al.*, 1983). Bellet *et al.* (1984) recently reported the specific radioimmunoassay method for hepatocellular carcinoma with use of monoclonal anti-AFP antibodies which were supposed to recognize certain unique epitopes of AFP. They have claimed that the method is more useful than conventional radioimmunoassays for the detection and monitoring of AFP-producing tumours in high risk populations, and for distinguishing hepatocellular carcinomas from nonmalignant liver diseases or healthy subjects.

We have recently found that the degree of fucosylation of AFP is a good marker for distinguishing hepatocellular carcinomas from nonmalignant liver diseases (Aoyagi *et al.*, 1984, 1985*a*, 1986). The fucosylated and non-fucosylated molecular species of AFP can be measured by crossed immuno-affinoelectrophoresis with LCA, by taking advantage of the reactivity of the fucosylated species with this lectin. Similar methods have been widely used for diagnosis of neural tube defects (Smith *et al.*, 1979; Toftager-Larsen *et al.*, 1983) and liver diseases (Miyazaki *et al.*, 1981; Breborowicz *et al.*, 1981) with lectins such as Con A and LCA. Recently a new attempt by immunoblotting technique for the discrimination of AFP species has been reported by Taketa *et al.* (1985).

However, the method of crossed immuno-affino-electrophoresis and antibody-affinity blotting has certain limitations as described above. Therefore, we aimed to provide a more convenient method which distinguishes between the fucosylated and non-fucosylated molecular species of AFP.

In the present study, we prepared monoclonal antibody 18H4 for such a purpose. The binding of 18H4 to the solid phase coated with the LCA-reactive species of AFP was inhibited by pretreating the solid phase with LCA (Figure 2b). Such inhibition was not observed when the solid phase was coated with the LCA-nonreactive species of AFP (Figure 2b). In similar experiments with monoclonal antibody 19F12, LCA only slightly inhibited the binding of this antibody to the solid phase coated with the LCA-reactive species of AFP (Figure 2a).

As described above, McAb18H4 was selected in the system with AFP-coated solid phase. Thereafter we developed a model system for the analysis of serum samples of AFP species. PcAb-coated polystyrene beads were prepared, and incubated with LCA-reactive or nonreactive (Figures 4a, b) species of AFP at various concentrations from 200 to 2,000 $ng\,ml^{-1}$. These AFP species trapped by solid phase PcAb were then treated with LCA, and incubated with $\beta$-D-galactosidase-labelled 18H4. The bound conjugate of 18H4 to AFP species was quantified, and % inhibition was calculated by comparison with the data from control experiments without LCA treatment. The binding of conjugate 18H4 to the LCA-reactive species of AFP trapped by solid phase PcAb was inhibited by treating with LCA (Figures 4a, b). Such inhibition was not obtained when the LCA-nonreactive species of AFP was examined in the system with PcAb-coated polystyrene beads and with LCA treatment (Figure 4b).

Inhibition of the binding of 18H4 to the LCA-reactive species of AFP can be explained by assuming that this monoclonal antibody recognizes an epitope of AFP which is closely located to the attachment site of a carbohydrate chain. (Pre)treatment with LCA of the LCA-reactive species of AFP would result in the binding of this lectin to its fucosylated sugar chain, and prevent 18H4 from binding to the AFP-molecular species by steric hindrance. The reason why complete inhibition was not achieved by the (pre)treatment with LCA may be that 18H4 does not recognize a carbohydrate chain itself.

### References

ABELEV, G.I. (1968). Production of embryonal serum α-globulin by hepatomas: Review of experimental and clinical data. *Cancer Res.*, **28**, 1344.

ALPERT, E. & FELLER, E.R. (1978). α-Fetoprotein (AFP) in benign liver disease. Evidence that normal liver regeneration does not induce AFP synthesis. *Gastroenterology*, **74**, 856.

ALPERT, M.E., URIEL, J. & DENECHAUD, B. (1968). Alpha$_1$ fetoglobulin in the diagnosis of human hepatoma. *N. Engl. J. Med.*, **278**, 984.

AOYAGI, Y., IKENAKA, T. & ICHIDA, F. (1977). Comparative chemical structures of human α-fetoprotein from fetal serum and from ascites fluid of a patient with hepatoma. *Cancer Res.*, **37**, 3663.

AOYAGI, Y., IKENAKA, T. & ICHIDA, F. (1978). Copper(II)-binding ability of human α-fetoprotein. *Cancer Res.*, **38**, 3483.

AOYAGI, Y., IKENAKA, T. & ICHIDA, F. (1979). α-Fetoprotein as a carrier protein in plasma and its bilirubin-binding ability. *Cancer Res.*, **39**, 3571.

AOYAGI, Y., ISEMURA, M., SUZUKI, Y. & 4 others. (1985*a*). Fucosylated α-fetoprotein as marker of early hepatocellular carcinoma. *Lancet*, **ii**, 1353.

AOYAGI, Y., ISEMURA, M., SUZUKI, Y. & 4 others. (1986). Change in fucosylation of α-fetoprotein on malignant transformation of liver cells. *Lancet*, **i**, 210.

AOYAGI, Y., ISEMURA, M., YOSIZAWA, Z. & 4 others. (1985*b*). Fucosylation of serum α-fetoprotein in patients with primary hepatocellular carcinoma. *Biochim. Biophys. Acta*, **830**, 217.

AOYAGI, Y., SUZUKI, Y., ISEMURA, M. & 6 others. (1984). Differential reactivity of α-fetoprotein with lectins and evaluation of its usefulness in the diagnosis of hepatocellular carcinoma. *Gann*, **75**, 809.

AOYAGI, Y., TAKAHASHI, Y., ODANI, S. & 3 others. (1982). Inhibitory effect of α-fetoprotein on protein synthesis in a reticulocyte lysate cell-free system. *J. Biol. Chem.*, **257**, 9566.

BAYARD, B. & KERCKAERT, J.-P. (1977). Characterization and isolation of nine rat alpha-fetoprotein variants by gel electrophoresis and lectin affinity chromatography. *Biochem. Biophys. Res. Commun.*, **77**, 489.

BELLET, D.H., WANDS, J.R., ISSELBACHER, K.J. & BOHUON, C. (1984). Serum α-fetoprotein levels in human disease: Perspective from a highly specific monoclonal radioimmunoassay. *Proc. Natl Acad. Sci.*, **81**, 3869.

BREBOROWICZ, J., MACKIEWICZ, A. & BREBOROWICZ, D. (1981). Microheterogeneity of alpha-fetoprotein in patient serum as demonstrated by lectin affino-electrophoresis. *Scand. J. Immunol.*, **14**, 15.

HEDIN, A., CARLSSON, L., BERGLUND, A. & HAMMARSTRÖM, S. (1983). A monoclonal antibody-enzyme immunoassay for serum carcinoembryonic antigen with increased specificity for carcinomas. *Proc. Natl Acad. Sci.*, **80**, 3470.

KATO, K., HAMAGUCHI, Y., FUKUI, H. & ISHIKAWA, E. (1976). Enzyme-linked immunoassay, conjugation of rabbit anti-(human immunoglobulin G) antibody with β-D-galactosidase from *Escherichia coli* and its use for human immunoglobulin G assay. *Eur. J. Biochem.*, **62**, 285.

KURMAN, R.J., SCARDINO, P.T., McINTIRE, K.R., WALDMANN, T.A. & JAVADPOUR, N. (1977). Cellular localization of alpha-fetoprotein and human chorionic gonadotropin in germ cell tumors of the testis using an indirect immunoperoxidase technique. *Cancer*, **40**, 2136.

LEHMANN, F.-G. (1976). Prognostic significance of alpha$_1$-fetoprotein in liver cirrhosis: Five-year prospective study. In *Oncodevelopmental Gene Expression*, Fishman, W. & Sell, S. (eds) p. 407. Academic Press Inc.: New York.

MACKIEWICZ, A. & BREBOROWICZ, J. (1981). Three-Dimensional affinity electrophoresis of human alpha-fetoprotien. In *Lectins-Biology, Biochemistry, Clinical Biochemistry*, Bøg-Hansen, T.C. (ed) Vol. **1**, p. 315. Walter de Gruyter: Berlin.

MIYAZAKI, J., ENDO, Y. & ODA, T. (1981). Lectin affinities of alpha-fetoprotein in liver cirrhosis, hepatocellular carcinoma and metastatic liver tumor. *Acta Hepatol. Jpn*, **22**, 1559.

NISHI, S. & HIRAI, H. (1973). Radioimmunoassay of α-fetoprotein in hepatoma, other liver diseases, and pregnancy. *Gann Monogr.*, **14**, 79.

OKUDA, K. & THE LIVER CANCER STUDY GROUP OF JAPAN. (1980). Primary liver cancers in Japan. *Cancer*, **45**, 2663.

ROUSLAHTI, E., SALASPURO, M., PIHKO, H., ANDERSSON, L. & SEPPÄLÄ, M. (1974). Serum α-fetoprotein: Diagnostic significance in liver disease. *Br. Med. J.*, **2**, 527.

ROUSLAHTI, E. & SEPPÄLÄ, M. (1979). α-Fetoprotein in cancer and fetal development. *Adv. Cancer Res.*, **29**, 275.

SMITH, C.J., KELLEHER, P.C., BÉLANGER, L. & DALLAIRE, L. (1979). Reactivity of amniotic fluid alpha-fetoprotein with concanavalin A in diagnosis of neural tube defects. *Br. Med. J.*, **1**, 920.

SMITH, C.J., MORRIS, H.P. & KELLEHER, P.C. (1977). Concanavalin A affinity molecular variants of α-fetoprotein in neonatal rat serum and in the serum of rats bearing hepatomas. *Cancer Res.*, **37**, 2651.

SMITH, J.B. (1970). Alpha-fetoprotein: Occurrence in certain malignant diseases and review of clinical applications. *Med. Clin. N. Am.*, **54**, 797.

TAKETA, K., ICHIKAWA, E., TAGA, H. & HIRAI, H. (1985). Antibody-affinity blotting, a sensitive technique for the detection of α-fetoprotein separated by lectin affinity electrophoresis in agarose gels. *Electrophoresis*, **6**, 492.

TAKETA, K., IZUMI, M. & ICHIKAWA, E. (1983). Distinct molecular species of human α-fetoprotein due to differential affinities to lectins. *Ann. N. Y. Acad. Sci.*, **417**, 61.

TOFTAGER-LARSEN, K., KJAERSGAARD, E. & NØRGAARD-PEDERSEN, B. (1983). Comparison of amniotic fluid alpha-fetoprotein reactivity to *Lens culinaris* agglutinin and concanavalin A in crossed-affinity immuno-electrophoresis: Ancillary test in the prenatal diagnosis of several fetal malformations. *Clin. Chem.*, **29**, 21.

WANDS, J.R., MARCINIAK, R.A., ISSELBACHER, K.J. & 4 others. (1982). Demonstration of previously undetected hepatitis B viral determinants in an Australian aboriginal population by monoclonal anti-HBs antibody radioimmunoassays. *Lancet*, **i**, 977.